# Evaluating GWAS-Identified SNPs for Age at Natural Menopause among Chinese Women

**DOI:** 10.1371/journal.pone.0058766

**Published:** 2013-03-25

**Authors:** Chong Shen, Ryan J. Delahanty, Yu-Tang Gao, Wei Lu, Yong-Bing Xiang, Ying Zheng, Qiuyin Cai, Wei Zheng, Xiao-Ou Shu, Jirong Long

**Affiliations:** 1 Division of Epidemiology, Department of Medicine, Vanderbilt Epidemiology Center, and Vanderbilt-Ingram Cancer Center, Vanderbilt University Medical Center, Nashville, Tennessee, United States of America; 2 Visiting from the School of Public Health, Nanjing Medical University, Nanjing, China; 3 Department of Epidemiology, Shanghai Cancer Institute, Shanghai, China; 4 Shanghai Municipal Center for Disease Prevention and Control, Shanghai Institute of Preventive Medicine, Shanghai, China; Peninsula College of Medicine and Dentistry, United Kingdom

## Abstract

**Background:**

Age at natural menopause (ANM) is a complex trait with high heritability and is associated with several major hormonal-related diseases. Recently, several genome-wide association studies (GWAS), conducted exclusively among women of European ancestry, have discovered dozens of genetic loci influencing ANM. No study has been conducted to evaluate whether these findings can be generalized to Chinese women.

**Methodology/Principal Findings:**

We evaluated the index single nucleotide polymorphisms (SNPs) in 19 GWAS-identified genetic susceptibility loci for ANM among 3,533 Chinese women who had natural menopause. We also investigated 3 additional SNPs which were in LD with the index SNP in European-ancestry but not in Asian-ancestry populations. Two genetic risk scores (GRS) were calculated to summarize SNPs across multiple loci one for all SNPs tested (GRS*all*), and one for SNPs which showed association in our study (GRS*sel*). All 22 SNPs showed the same association direction as previously reported. Eight SNPs were nominally statistically significant with *P*≤0.05: rs4246511 (*RHBDL2*), rs12461110 (*NLRP11*), rs2307449 (*POLG*), rs12611091 (*BRSK1*), rs1172822 (*BRSK1*), rs365132 (*UIMC1*), rs2720044 (*ASH2L*), and rs7246479 (*TMEM150B*). Especially, SNPs rs4246511, rs365132, rs1172822, and rs7246479 remained significant even after Bonferroni correction. Significant associations were observed for GRS. Women in the highest quartile began menopause 0.7 years (*P* = 3.24×10^−9^) and 0.9 years (*P* = 4.61×10^−11^) later than those in the lowest quartile for GRS*sel* and GRS*all*, respectively.

**Conclusions:**

Among the 22 investigated SNPs, eight showed associations with ANM (P<0.05) in our Chinese population. Results from this study extend some recent GWAS findings to the Asian-ancestry population and may guide future efforts to identify genetic determination of menopause.

## Introduction

The occurrence and timing of menarche and menopause play major roles in a woman's life and reproduction-related events. Menopause is the final reproductive event and unambiguously marks the ovarian aging and exhaustion of the primordial follicle pool in women [1]. Menopause at a younger age often predicts an earlier induction of subfertility, sterility, and transition to cycle irregularity and vice versa [2].

Generally, age at natural menopause (ANM) ranges between 40–60 years with a median of 49 to 52 years, depending on the population [3]. Population-based studies report that the beginning of transition from regular to irregular menstrual cycles is 6–7 years ahead of ANM. This probably reflects the end of female fertility, which is nearly 10 years ahead of ANM [4]. Menopause age variation influences the levels of serum estrogen, follicle stimulating hormone, and progesterone, all which affect the well-being of women. Series studies report that the variation of menopause is associated with several major age-related diseases [5], such as cardiovascular disease [6], [7], breast cancer [8], osteoporosis [9], and depression [10]. So, identifying factors which determine variations of ANM may provide insights into the pathogenesis of these diseases.

ANM is a complex trait, influenced by multiple environmental and genetic factors [11]. ANM has high heritability (70%). Genome-wide association studies (GWAS) have identified multiple genetic loci which influence ANM [12]–[16]. All of these GWAS were exclusively conducted in European-ancestry populations. Given the considerable differences in genetic architecture including allele frequencies, linkage disequilibrium (LD) structure and genetic diversity across ethnic groups, it is important to investigate whether GWAS-identified variants are associated with ANM in non-European populations. In this current study, we report a systematic evaluation of the GWAS-identified loci in association with ANM among Chinese.

## Methods

### Study Participants and Data Collection

This analysis included women from Shanghai, China, who were participants in multiple ongoing GWAS: the Shanghai Breast Cancer Genetics Study (SBCGS) [17], the Shanghai Endometrial Cancer Genetics Study (SECGS) [18], the Shanghai Diabetes Genetics Study (SDGS) [19], the Shanghai Colorectal Cancer Genetics Study [20], the Upper GI Cancer Genetics Study, and the Panscan Cancer Genetics Study [21]. Among these studies, 4,106 reported detailed information of menopause. We excluded 573 women including 390 individuals whose menopause were disease-related, 158 individuals who took hormone replacement treatment, and 25 individuals with other non-nature cause menopause. Thus, 3,533 post-menopausal women were finally included in this study.

All parent studies are population-based and similar study protocols were used across studies to collect blood or buccal cell samples and relevant exposure information. Interviews were conducted in-person, and structured epidemiological questionnaires were administered by trained interviewers. Age at natural menopause (ANM) is defined as the age when menstrual periods ceased permanently and naturally (in years). Anthropometric characteristics including birth date, education, occupation, and age at menarche were collected in addition to information regarding individual habits, history of hormone replacement therapy, prior use of oral contraceptives, and post-disease status. Written, informed consent was obtained from all participants prior to interview, and study protocols were approved by the institutional review boards of all institutions involved in the study.

### SNP Selection and Genotyping

To date, 28 SNPs have been reported to be associated with ANM in four GWASs with *P*<5×10^−8^ among European-ancestry women [12]–[15]. These SNPs represent 19 independent loci with low pairwise linkage disequilibrium (LD) (r^2^<0.2) based on the HapMap Asian data (Table S1). We also searched flanking 500kb of each of the 28 index SNPs using the HapMap data. There were three common SNPs (MAF>0.01) which are in strong LD (r^2^≥0.8) with the index SNP in European-ancestry, but not in Asian-ancestry populations (r^2^<0.2). Three such SNPs, rs4667673, rs2517388, and rs12294104, were also included in this study (Table S1).

Genotyping of all participants was performed using Affymetrix SNP Array 6.0 [19], Illumina Omniexpress [22], Illumina 550 [22], Illumina 660. Detailed quality control (QC) procedures were described in these studies. In general, we removed SNPs with minor allele frequency (MAF) less than 5%, Hardy-Weinberg *P-*values <1×10^−5^, samples with >5% missing genotypes, inconsistent sex between self-reported and genotype based, non-Asian subjects based on clustering with HapMap subjects, and close-relationship subjects based on identity-by-descent (IBD) analyses.

For SNPs which were not directly genotyped through the GWAS chip, imputed data were extracted. Using the Han Chinese (CHB) and Japanese (JPT) data from HapMap Phase 2 (release 24) and Phase 3 (release 2) as the references, we used MACH 1.0 software (http://www.sph.umich.edu/csg/abecasis/MaCH/) to conduct imputation within each GWAS. The imputation quality RSQR was more than 0.91 for all SNPs included in the present study, with the exceptions of rs7246479 (0.79) and rs2277339 (0.77). Dosage data (with imputation uncertainty taken into account) were investigated in relation to ANM.

### Statistical Analysis

ANM was presented as the means ± SD between different groups. Student’s t-test and analysis of variance (ANOVA) were applied to evaluate the differences of ANM by non-genetic factors. Linear regression was used to derive beta coefficients and 95% CIs for associations between genotypes and ANM under the additive model. All regression models include adjustment for birth cohort, education, and disease status. *P*-values reported for single SNPs are one-tailed since the hypothesis tested is whether the direction of association was consistent between Asian-ancestry and European-ancestry women. Program Quanto (http://hydra.usc.edu/gxe/) was used to estimate statistical power.

The binomial sign test was conducted to assess the consistency of the associations previously reported and seen in our study. Genetic risk scores (GRS) were used to summarize multiple SNPs [23], [24]. Two genetic risk scores were constructed in current study. The GRS*sel* included 8 SNPs which were associated with ANM (*P*<0.05) in our study; the GRS*all* included all 22 SNPs evaluated. Relationships between GRS quartiles and ANM were evaluated by linear regression and ANOVA. All statistical analyses were conducted using SAS, version 9.2 (SAS Institute, Inc., Cary, N.C.).

## Results

Table 1 presents the mean ANM by education, occupation, number of live births, use of oral contraceptives, age at menarche, and disease status. Significant differences of ANM were observed across each of these categories with the exception of age at menarche.

**Table 1:** Characteristics of study participants.

Variable	Category	N	*Age at natural menopause*			
			Mean	*SD*	*P* a	*P* b
Education					<0.001	
	non	643	49.39	4.04		
	Elementary	725	49.42	3.84		
	Middle school	1000	49.12	3.49		
	High school	757	49.71	3.42		
	High profession/College+	407	50.48	3.32		
Occupation					<0.001	<0.001
	Professional	937	50.07	3.40		
	Clerical	642	49.61	3.52		
	Manual workers	1952	49.22	3.77		
Number of live birth						<0.001
	0	139	48.63	3.97		
	1	752	48.93	3.47		
	2	1261	49.90	3.35		
	3	772	49.66	4.09		
	4+	609	49.43	3.71		
Ever take oral contraceptives					<0.001	
	No	869	49.79	3.67		
	Yes	278	50.64	3.47		
Age at menarche (years)					0.902	0.7179
	≤12	237	49.62	4.00		
	13	588	49.62	3.38		
	14	611	49.59	3.52		
	15	733	49.47	3.75		
	16	638	49.48	3.30		
	17	398	49.46	3.94		
	≥18	326	49.32	4.21		
Disease					<0.001	0.9861
	Healthy controls	1308	49.20	3.77		
	Breast cancer	850	49.86	3.63		
	Endometrial cancer	427	50.42	3.41		
	Colorectal cancer	322	49.20	3.46		
	Upper GI cancer	160	49.0	3.76		
	Type 2 diabetes	466	49.31	3.46		

a *P* for ANOVA.

b *adjusting for Education.*

Table 2 shows the results of SNPs included in this study. 22 SNPs showed the same direction of association as previously reported (binomial sign test *P*<1×10^−4^). Eight SNPs were nominally significant with *P*≤0.05: rs4246511 (*RHBDL2*), rs365132 (*UIMC1*), rs2307449 (*POLG*), rs1246111 (*NLRP11*), rs12611091 (*BRSK1*), rs1172822 (*BRSK1*), rs2720044 (*ASH2L*), and rs7246479 (*TMEM150B*). Notably, SNPs rs4246511 (*RHBDL2*), rs365132 (*UIMC1*), rs1172822 (*BRSK1*), and rs7246479 (*TMEM150B*) showed associations with P≤0.002 and remained significant even after Bonferroni correction. The per-allele effect on ANM was 0.287, 0.284, 0.600, 0.487 years for rs4246511, rs365132, rs1172822, and rs7246479. These estimates are comparable with previous results in European-ancestry women. No significant associations were found for the other 14 SNPs. Among them, three have a MAF<0.05, including rs4886238, rs7123626, and rs7333181. Considering the disease of the subjects could also show overlap in the genetic architecture with menopausal age, the association analysis in the healthy controls was further performed as a sensitivity analysis and the results indicated that six SNPs of the above eight SNPs associated with ANM still showed association with P<0.05 (Table S2).

**Table 2:** Association results of 16 SNPs and age of natural menopause in Chinese women.

SNPs	Chr^d^	Position	Nearest gene	Allele	Reported studies			Shanghai population				
				Reference/Effect	EAF^e^	B^f^	P	EAF^e^	B f	P	Power	GRS*sel* g
rs4246511	1	39152972	*RHBDL2*	C/T	0.187	0.240	1.02×10^−16^	0.612	0.287	**0.002**	0.879	Y
rs1635501	1	240107398	*EXO1*	T/C	0.482	−0.164	8.46×10^−10^	0.235	−0.019	0.851	0.056	
rs2303369	2	27568920	*FNDC4*	C/T	0.389	−0.175	8.40×10^−14^	0.139	−0.094	0.455	0.106	
rs10183486	2	171699217	*TLK1*	C/T	0.357	−0.196	2.21×10^−14^	0.070	−0.166	0.325	0.165	
rs4667673^a^	2	171551661	*TLK1*	C/G	0.375			0.386	0.144	0.121	0.353	
rs4693089	4	84592646	*HEL308*	A/G	0.488	0.228	1.80×10^−19^	0.658	0.111	0.213	0.231	
rs365132	5	176311180	*UIMC1*	G/T	0.491	0.39	2.60×10^−12^	0.504	0.284	**0.001**	0.869	Y
rs1046089	6	31710946	*BAT2*	G/A	0.354	−0.213	1.45×10^−59^	0.336	−0.075	0.400	0.123	
rs2153157	6	11005474	*SYCP2L*	G/A	0.491	0.165	7.76×10^−12^	0.677	0.166	0.075	0.412	
rs2517388	8	38096889	*ASH2L*	T/G	0.178	0.262	2.47×10^−19^	0.662	0.071	0.445	0.123	
rs2720044^b^	8	38099744	*ASH2L*	A/C	0.188			0.265	0.225	**0.029**	0.610	Y
rs12294104	11	30339475	*MPPED2*	C/T	0.174	0.225	2.25×10^−12^	0.102	0.060	0.702	0.073	
rs7123626^c^	11	30282441	*FSHB*	G/A	0.112			0.036	0.085	0.715	0.064	
rs2277339	12	55432336	*PRIM1*	T/G	0.103	−0.380	3.56×10^−13^	0.274	−0.164	0.136	0.381	
rs4886238	13	60011740	*TDRD3*	G/A	0.334	0.170	9.31×10^−15^	0.038	0.013	0.955	0.050	
rs7333181	13	111019298	*SOX1*	G/A	0.120	0.520	2.38×10^−19^	0.038	−0.135	0.558	0.087	
rs2307449	15	87664932	*POLG*	T/G	0.400	−0.184	9.53×10^−11^	0.341	−0.211	**0.019**	0.634	Y
rs10852344	16	11924420	*COX6CP1*	T/C	0.482	0.168	1.01×10^−11^	0.847	0.188	0.130	0.356	
rs12461110	19	61012475	*NLRP11*	G/A	0.326	−0.158	8.74×10^−10^	0.293	−0.201	**0.034**	0.558	Y
rs12611091	19	60492141	*BRSK1*	T/C	0.480	0.330	1.10×10^−10^	0.773	0.285	**0.008**	0.767	Y
rs1172822	19	60511657	*BRSK1*	C/T	0.390	−0.49	2.50×10^−8^	0.095	−0.600	**6.64×10** ^−**5**^	0.978	Y
rs7246479	19	60516144	*TMEM150B*	G/T	0.480	0.360	2.90×10^−12^	0.815	0.487	**3.75×10** ^−**5**^	0.993	Y

a In LD with GWAS-identified SNP rs10183486 in CEU (r^2^ = 0.863) but not in CHB (r^2^ = 0.005).

b In LD with GWAS-identified SNP rs2517388 in CEU (r^2^ = 0.843) but not in CHB (r^2^ = 0.169).

c In LD with GWAS-identified SNP rs12294104 in CEU (r^2^ = 0.877) but not in CHB (r^2^ = 0.070).

d Chr: chromosome.

e EAF: effect allele frequency.

f Partial regression coefficient was calculated for years per allele change in ANM (years).

g GRS: genetic risk score, Significant SNPs were selected in the calculation of the GRS*sel*.

GRS analyses also showed significant associations (Table 3). A dose-response association was observed between the number of effect alleles and ANM. The average ANM across increasing quartiles of the GRS*sel* were 49.21, 49.34, 49.60 and 49.90 (*P* = 3.24×10^−9^). Similar association was observed for GRS*all* with women in the highest quartile starting menopause approximately 0.9 years later than those in the lowest quartile (*P* = 4.61×10^−11^).

**Table 3:** Association analysis of GRS and ANM.

*GRSsel* quartile	N	ANM	*GRSall* quartile	N	ANM
		(years, mean ± SD)			(years, mean ± SD)
Q1 (<8.93)	892	49.21±3.78	Q1 (<24.45)	881	49.00±3.72
Q2 (8.93–9.96)	869	49.34±3.55	Q2 (24.45–26.45)	885	49.48±3.62
Q3 (9.97–11.06)	888	49.60±3.61	Q3 (26.46–28.19)	877	49.69±3.41
Q4 (≥11.07)	884	49.90±3.65	Q4 (≥28.20)	890	49.87±3.80
*P* for trend		3.24×10^−9^	*P* for trend		4.61×10^−11^

## Discussion

In this study, we evaluated 22 SNPs in GWAS-identified genetic loci for menopause age originally discovered in Europeans in our Chinese population. Significant associations were observed for 8 SNPs and association directions were all same as originally reported. Furthermore, six of eight SNPs were replicated to be correlated with ANM in the healthy controls. Of the 8 replicated SNPs, four were located in 19q13.42, including rs12461110 at exon 5 of *NLRP11*gene, rs7246479 at exon 8 of *TMEM150B* gene, rs1172822 and rs12611091 at introns 19 and 5, respectively, of the *BRSK1* gene. SNP rs12461110 results in a missense change from Proline to Leucine. The *NLRP11* gene was reported to be involved in multiprotein complexes called inflammasomes and implicated in the activation of proinflammatory caspases [25]. SNP rs7246479 results in amino acid change from Leucine to Phenylalanine alanine. The *TMEM150B* gene encodes transmembrane protein 150B, which belongs to the DRAM/TMEM150 family and is involved in apoptosis pathway. SNP rs1172822 may affect the binding of the transcription factor CdxA and C/EBP binding sites (FASTSNP, http://fastsnp.ibms.sinica.edu.tw), whereas rs12611091 has no known or predicted function. *BRSK1* is highly expressed in the human brain and moderately expressed in mammalian ovaries [26]. The *BRSK1* was previously published by multiple studies in European ancestry populations and was recently replicated in Hispanic women [27]. Further functional exploration may shed insight to the role of the *BRSK1* gene or other genes impacting menopause.

SNP rs4246511 is located at intron 3 of the *RHBDL2* gene, which may function as an intramembrane serine protease [28]. SNP rs2307449 is located at intron 18 of the *POLG* gene, which encodes the catalytic subunit of mitochondrial DNA polymerase. The enzyme is responsible for replication and repair of mitochondrial DNA, and modulates the balance between DNA synthesis and excision [29], [30]. For the *ASH2L* gene, the previously reported SNP, rs2517388, did not replicate in our study. However, the other SNP rs2720044, which is in strong LD with rs2517388 in European-ancestry population with r^2^ = 0.843 but not in Asian-ancestry with r^2^ = 0.169, was significantly associated with ANM in our population. These results indicate that the *ASH2L* gene might harbor different susceptible variants to ANM across ethnic groups. The *ASH2L* is required during early embryogenesis [31] and recruited to the inactive X chromosome at the onset of stable X inactivation [32].

We did not replicate the association of other 14 SNPs with ANM. Similarly, in a recent study in Hispanic women, only two of 14 evaluated menopause loci were replicated [27]. For the 14 non-replicated SNPs, we have limited statistical power (<40%) to detect evidence of association. Allele frequency is different across ethnic groups, which may affect the confirmation. Among the 10 non-replicated SNPs, three showed very low MAF in our study. In addition, LD structure often differs across populations, and GWAS hits are typically markers in LD with functional alleles. Therefore, it is expected that many genetic variants identified in one population cannot be directly replicated in other populations. In addition, the effect size for the same SNP may differ across ethnic groups due to different environment exposure. This may also result in the non-replication of the SNPs in other ethnic groups. Furthermore, replication study with larger sample size would be warranted.

Strengths of the study include the focus on replicating significant GWAS-identified SNPs among Asian women and a relatively large sample size. Furthermore, we investigated SNPs in LD with the SNPs reported in European-ancestry but not in Asian-ancestry populations, which would enhance the genomic coverage of susceptible regions for ANM. Results observed in the present study should be interpreted considering several limitations. First, GWAS-identified SNPs were selected only if they met a GWAS significance threshold (*P*<5×10^−8^), and some SNPs with less significant *P*-values may be ignored. Second, different studies used different genotyping platforms, and there are only a small portion of SNPs overlapping between these platforms. Some SNPs were directly genotyped in some parent studies but imputed in other parent studies. Third, no surrogate SNP was identified for rs16991615 and rs236114, and we did not investigate these two loci. Fourth, ANM was self-reported with measured error, although this error is unlikely to be associated with genotype.

In summary, we successfully replicated eight SNPs associated with age at natural menopause in Chinese women. This is the first report of the common shared genetic architecture affecting menopause between European and Chinese women. Further research on these genetic regions will enhance our understanding of the genetic determination of menopause.

## Supporting Information

Table S1 **GWAS-identified SNPs for ANM.**
(DOC)

Table S2 **Association results of 22 SNPs and ANM in Healthy population.**
(DOCX)
