# Supplementary material for: Evaluating GWAS-Identified SNPs for Age at Natural Menopause among Chinese Women
Source: PLoS One. 2013 Mar 25;8(3):e58766. doi: 10.1371/journal.pone.0058766 (PMC3607593; doi:10.1371/journal.pone.0058766)
Supplement: Table S1 — GWAS-identified SNPs for ANM. (DOC) [file pone.0058766.s001.doc]

Table S1. GWAS-identified SNPs for ANM

| SNP | Proxy | Chromosome | Distance (bp) | r^2^ (Caucasian) | r^2^ (Asian) |
| --- | --- | --- | --- | --- | --- |
| rs4246511 ^b^ |  | 1 |  |  |  |
| rs1635501 ^b^ |  | 1 |  |  |  |
| rs2303369 ^b^ |  | 2 |  |  |  |
| rs10183486 ^b^ | rs4667673 ^b,c^ | 2 | 147556 | 0.863 | 0.005 |
|  |  |  |  |  |  |
|  | rs7421377 ^a,c^ | 2 | 5869 | 0.93 | 0.03 |
|  | rs10171850 ^a,c^ | 2 | 15770 | 0.93 | 0.029 |
|  | rs13410683 ^a,c^ | 2 | 17249 | 0.897 | 0.069 |
|  | rs12477649 ^a,c^ | 2 | 28295 | 0.897 | 0.07 |
|  | rs930036 ^a,c^ | 2 | 49953 | 0.897 | 0.028 |
|  | rs930035 ^a,c^ | 2 | 49996 | 0.897 | 0.069 |
|  | rs964172 ^a,c^ | 2 | 55242 | 0.897 | 0.069 |
|  | rs4667673 ^a,c^ | 2 | 147556 | 0.863 | 0.066 |
|  | rs4668354 ^a,c^ | 2 | 176221 | 0.83 | 0.067 |
|  | rs3770438 ^a,c^ | 2 | 185186 | 0.83 | 0.07 |
|  | rs11902369 ^a,c^ | 2 | 222020 | 0.799 | 0.047 |
| rs4693089 ^b^ | - | 4 |  |  |  |
| rs365132 ^b^ | rs7718874 ^a^ | 5 | 20509 | 0.967 | 1 |
|  | rs402511 ^a^ | 5 | 55866 | 0.934 | 0.978 |
|  | rs691141 ^a^ | 5 | 55276 | 0.759 | 0.811 |
| rs1046089 ^b^ |  | 6 |  |  |  |
| rs2153157 ^b^ |  | 6 |  |  |  |
| rs2517388 ^b^ | rs2720044 ^b,c^ | 8 | 2855 | 0.843 | 0.169 |
| rs12294104 ^b^ | rs7123626 ^b,c^ | 11 | 57034 | 0.92 | 0.07 |
| rs2277339 ^b^ | - | 12 |  |  |  |
| rs4886238 ^b^ | - | 13 |  |  |  |
| rs7333181 ^b^ | - | 13 |  |  |  |
| rs2307449 ^b^ | - | 15 |  |  |  |
| rs10852344 ^b^ |  | 16 |  |  | 0.157 |
| rs1172822 ^b^ | rs11668344 ^a^ | 19 | 13819 | 0.894 | 1 |
|  | rs2384687 ^a^ | 19 | 11343 | 0.768 | 1 |
|  | rs897798 ^a^ | 19 | 13909 | 0.683 | 1 |
|  | rs7246479 ^b^ | 19 | 4487 | 0.558 | 0.272 |
|  | rs1551562 ^a^ | 19 | 4964 | 0.520 | 0.272 |
|  | rs12611091 ^b^ | 19 | 19516 | 0.447 | 0.092 |
| rs12461110 ^b^ |  | 19 |  |  |  |
| rs12611091 ^b^ | rs897798 ^a^ | 19 | 33425 | 0.642 | 0.364 |
|  | rs7246479 ^b^ | 19 | 24003 | 0.538 | 0.092 |
|  | rs1172822 ^b^ | 19 | 19516 | 0.447 | 0.092 |
|  | rs11668344 ^a^ | 19 | 33335 | 0.383 | 0.092 |
|  | rs2384687 ^a^ | 19 | 30859 | 0.292 | 0.092 |
|  | rs1551562 ^a^ | 19 | 14552 | 0.267 | 0.072 |
| rs7246479 ^b^ | rs897798 ^a^ | 19 | 9422 | 0.818 | 0.364 |
|  | rs2384687 ^a^ | 19 | 6856 | 0.622 | 0.272 |
|  | rs1172822 ^b^ | 19 | 4487 | 0.558 | 0.272 |
|  | rs11668344 ^a^ | 19 | 9332 | 0.538 | 0.272 |
|  | rs12611091 ^b^ | 19 | 24003 | 0.538 | 0.272 |
|  | rs1551562 ^a^ | 19 | 9451 | 0.29 | 0.243 |
| rs16991615 ^a^ | - | 20 |  |  |  |
| rs236114 ^a^ | - | 20 |  |  |  |

a. Excluded in the present study.

b. Included in the present study.

c. In LD with index SNP in Europeans (r^2^≥0.8) but not in Asians (r^2^<0.2).
