# Supplementary material for: Evaluating GWAS-Identified SNPs for Age at Natural Menopause among Chinese Women
Source: PLoS One. 2013 Mar 25;8(3):e58766. doi: 10.1371/journal.pone.0058766 (PMC3607593; doi:10.1371/journal.pone.0058766)
Supplement: Table S2 — Association results of 22 SNPs and ANM in Healthy population. (DOCX) [file pone.0058766.s002.docx]

Table S2. Association results of 22 SNPs and ANM in Healthy population

| SNP | Estimate | Standard Error |  | P value |
| --- | --- | --- | --- | --- |
| rs4246511 | 0.5549 | 0.1541 |  | **0.0003** |
| rs1635501 | 0.0558 | 0.1730 |  | 0.7470 |
| rs2303369 | 0.2115 | 0.2121 |  | 0.3189 |
| rs10183486 | 0.0762 | 0.2764 |  | 0.7829 |
| rs4667673 | 0.0980 | 0.1579 |  | 0.5349 |
| rs4693089 | 0.2114 | 0.1484 |  | 0.1545 |
| rs365132 | 0.4490 | 0.1503 |  | **0.0029** |
| rs1046089 | -0.1857 | 0.1517 |  | 0.2212 |
| rs2153157 | 0.1866 | 0.1597 |  | 0.2427 |
| rs2517388 | 0.0691 | 0.1568 |  | 0.6594 |
| rs2720044 | 0.2727 | 0.1761 |  | 0.1217 |
| rs12294104 | -0.1234 | 0.2648 |  | 0.6411 |
| rs7123626 | 0.2149 | 0.3941 |  | 0.5857 |
| rs2277339 | -0.0431 | 0.1841 |  | 0.8147 |
| rs4886238 | -0.4055 | 0.3917 |  | 0.3007 |
| rs7333181 | 0.0999 | 0.3783 |  | 0.7917 |
| rs2307449 | -0.3918 | 0.1489 |  | **0.0086** |
| rs10852344 | 0.0451 | 0.2177 |  | 0.8359 |
| rs12461110 | -0.0714 | 0.1619 |  | 0.6595 |
| rs12611091 | 0.5291 | 0.1855 |  | **0.0044** |
| rs1172822 | -0.8113 | 0.2684 |  | **0.0026** |
| rs7246479 | 0.5588 | 0.1989 |  | **0.0050** |
